# Commentary: Two Ways of Thinking About Self-Control

**DOI:** 10.3389/fpsyg.2021.718715

**Published:** 2021-07-21

**Authors:** Meysam Moayery

**Affiliations:** Department of Marketing, Faculty of Economics and Business Administration, Vilnius University, Vilnius, Lithuania

**Keywords:** self-control, self-monitoring failures, ego-depletion, reflective and impulsive determinants, non-conscious self-control

## Introduction

The basic premise of Vosgerau et al.'s (2020; henceforth VSH) article is one that few consumer behavior researchers and psychologists would debate: “self-control conflicts are subjective” and “not all consumers pursue the same superordinate long-term goals” (p. 187). While the dominant paradigm defines self-control as a consumer's choice to desist from hedonic consumption, VSH outline self-control failures as choices violating superordinate long-term goals (whether hedonic or utilitarian) that entail the anticipation of regret. Therefore, the central message of VSH's framework is that self-control does not require abstinence from pleasure (i.e., exerting self-control ≠ sacrificing pleasure). VSH also argue that this conceptualization is vital for the construct validity of self-control studies in consumer research.

This commentary agrees with the idea that the choice of hedonic consumption (e.g., chocolate cake) is not always equated with failures in self-control. In this regard, Moayery et al. (2019b) showed that if consumers do not endorse any standards regarding their diet, they might buy more unhealthy snacks even when enough self-regulatory resources are available. Similarly, it has been suggested that the desire for unhealthy food cannot be translated into temptation, unless the person follows a healthy diet (Hofmann and Kotabe, 2012). At the same time, in this paper, I wish to draw attention to three issues that help in making the distinction between the perspective presented by VSH and my perspective on self-control. These issues are self-monitoring failures, ego-depletion effects, and reflective and impulsive determinants of self-control. Therefore, building on VSH's framework, this commentary proposes an expanded, more inclusive perspective on self-control problems. In order to flesh out this idea, I attempt to (1) clarify that people often fail to detect a conflict of self-control because they fail to self-monitor, (2) challenge the idea that ego-depletion is not a real phenomenon, and (3) highlight the role of reflective and impulsive aspects of self-control. This commentary also discusses a direction for future studies with emphasis on non-conscious forms of self-control and some methodological considerations for measuring impulses.

## Self-Monitoring Failures

VSH's framework considers the concept of opposing preferences as the core idea of the definition of self-control (i.e., a conflict between co-existing selves). These preferences may vary over time, together with a lack of symmetry in their importance. Therefore, according to this framework, exerting self-control can be conceived as resolving a goal conflict (e.g., a health goal vs. immediate gratification) in service of superordinate long-term preferences. On the other hand, self-control failures are represented as yielding to temptation (i.e., the violation of superordinate long-term benefits) for which the consumer expects to feel regret. In the same vein, recent studies also provide evidence for a link between anticipated emotions and self-control judgments (Kotabe et al., 2019). However, this commentary wants to emphasize the fact that people are not always able to perceive self-control conflicts due to self-monitoring failures.

Self-monitoring is one the major ingredients of self-control. This includes keeping track of one's responses and actions to compare the real situation of the self to one's goals and ideals (i.e., recognition of conflicts; Baumeister and Heatherton, 1996; Baumeister, 2002). Simply stated, self-monitoring is closely related to the identification of an incompatibility between the expected outcomes of acting based on desire and the person's value system and self-regulatory goal standards (Hofmann and Kotabe, 2012). Therefore, conflict monitoring can be deemed as a fundamental cognitive function supporting the process through which control is recruited (Yeung, 2014). In this regard, prior research has shown that self-monitoring is associated with a wide range of behaviors including improved weight management, healthy dietary change (see Spring et al., 2020), and spending behaviors (for a review, see Moayery et al., 2019a). Nevertheless, a person may temporarily fail to experience a self-control conflict (i.e., self-monitoring failure; Hofmann and Kotabe, 2012). Indeed, sometimes communication to the higher cognitive system (in the prefrontal cortex), which is linked to self-control, is blocked due to consumers' lack of attention, which means that goals are kept in the pre-conscious domain in this situation (Plassmann and Mormann, 2017). For example, Mr. A, who is having a dinner date at a restaurant, may see a piece of chocolate cake on the dessert menu and be aware that his health goal is to eat healthy foods. However, he might not be paying attention toward his goal, and hence the absence of communication with the higher cognitive systems that are linked to self-control (see Plassmann and Mormann, 2017). This lack of attention can be partially attributed to the fact people often track multiple goals at the same time (Fujita, 2011; Neal et al., 2017). For instance, Mr. A has to pay attention to his date (e.g., presenting himself in a favorable manner) as well as his eating behavior (e.g., to prevent overeating non-healthy foods) simultaneously (see Fujita, 2011).

## Ego-Depletion Effects

VSH discuss that it is difficult to draw a generalizable conclusion from the current predominant paradigm for studying self-control due to the application of ego-depletion effects. They doubt the existence of ego-depletion and claim that it may be arguable to conjecture on what caused the effects that were observed in current studies on self-control. They argue that ego-depletion might be a result of cognitive fatigue or type-I errors. Although this commentary concurs with VSH that there are several challenges and criticisms of ego-depletion (for reviews, see Englert, 2016; André et al., 2019; Alquist et al., 2020), it appears premature to dismiss the phenomenon because none of the critical evidence provides conclusive answers that ego-depletion does not exist (Friese et al., 2019). In fact, the application of the ego-depletion theory in everyday problems, across a variety of domains, has been tested successfully. These include impulse buying, alcohol consumption, eating behavior, self-protective behavior, logical thinking, making choices, sport and exercise behavior, and even math test performance (Muraven et al., 2005; Vohs, 2006; Hofmann et al., 2007; Vohs and Faber, 2007; Bertrams et al., 2016; Englert, 2016; Moayery et al., 2019b). These findings are in line with the basic premise of the strength model emphasizing that different tasks and functions use a global resource (i.e., relying on a domain-general resource), which can become depleted by successive attempts at self-regulation (Bertrams et al., 2016; Englert, 2016; Wagner and Heatherton, 2016; Baumeister and Vohs, 2018). According to this account, the depleted state results in impaired self-control task performance, the phenomenon known as ego-depletion (Hagger et al., 2010; Baumeister et al., 2018; Alquist et al., 2020).

This commentary assumes that the ambiguity associated with ego-depletion is related to the uncertainties regarding the underlying mechanism as well as the nature of this limited resource (see Hedgcock et al., 2012; Friese et al., 2019). To address this concern, ample research has documented the cognitive, psychological and neurological aspects of ego-depletion as supporting evidence for this phenomenon (for reviews, see Englert, 2016; Wagner and Heatherton, 2016). For instance, it has been demonstrated that self-regulatory resource depletion weakens some kinds of cognitive activities (e.g., complex thinking) which need active guidance by the self (Schmeichel et al., 2003). Interestingly, Vohs et al. (2011), in line with the limited-resource model (i.e., the strength model), showed that ego-depletion is not equivalent to fatigue. They concluded that the ego-depletion effect appears when there is a lack of self-regulatory capacity, which can be interpreted as the tiredness of the inner energy that regulates unwanted responses. Another study also empirically demonstrated that self-regulatory resource depletion reduces the activity of the right middle frontal gyrus (located in the dorsolateral prefrontal cortex; Hedgcock et al., 2012). To conclude, although this commentary agrees that the ego-depletion theory suffers from several shortcomings (see Friese et al., 2019), up until now, alternative explanations cannot provide a complete picture of the self-control issue without subtly reintroducing the idea of depleted energies (Baumeister and Vohs, 2018; Baumeister et al., 2018). For example, in a move toward a new perspective on the nature of effortful control, Baumeister and his colleagues have recently integrated the resource models with other alternative theoretical approaches (e.g., cost-benefit models; Alquist et al., 2020; see also André et al., 2019). This body of literature provides new insight into the true nature of the capacity or resource to exert effortful control and related fatigue-like effects.

## Reflective and Impulsive Determinants of Self-Control

It can be discussed that VSH's framework ignores reflective self-control conflicts due to overemphasizing consumption self-control (e.g., consumption of time, money, and food), which makes it difficult to generalize the model to other behavior domains with more serious consequences (see Lamberton, 2020). This commentary assumes that making deliberate judgments and evaluations, as well as suppressing impulses are executive functions of this higher-order mental operation, known as the “reflective system” (Hofmann et al., 2009). This reflective system can be dietary restraint standards concerning eating or purchasing snacks (Hofmann et al., 2007; Moayery et al., 2019b), reflective trust in close relationships (Murray et al., 2011), or action and coping plans in health care professional behavior (Presseau et al., 2014), etc.

Now imagine a conflict between Sally and her partner, Harry: Sally likes her house to be clean, but it is not one of Harry's priorities. While Sally needs Harry's help to achieve her goal of keeping things clean, asking him to cooperate means she is left vulnerable to his non-responsiveness. If Sally wants to ask for his help (i.e., ignoring her need to avoid his rejection), she needs to make sure that it is safe for her to depend on Harry to achieve her goals (Murray et al., 2011). Are deliberate or conscious expectations of partner caring (i.e., reflective trust) enough to govern self-protection in romantic relationships? According to Murray et al. (2011), impulsive trust (i.e., automatic evaluative associations to a partner) and reflective trust (i.e., relatively conscious) jointly regulate self-protection in close relationships. Applied to eating behavior, prior research has shown that the joint consideration of impulsive (e.g., an implicit measure) and reflective influences (e.g., dietary restraint), as well as self-regulatory resources may help to predict unhealthy snack intakes more accurately (Hofmann et al., 2007; Friese et al., 2008). This concurs with findings from other studies in a representative range of self-control domains including drinking, impulse buying, sexual interest behavior, and other social interactions (see Hofmann et al., 2009; Moayery et al., 2019b). Taken together, these results suggest that a joint function of reflective and impulsive mechanisms can predict most human behaviors, with special application to consumer and health psychology (Strack et al., 2006; Hofmann et al., 2008a). According to this logic, “when consumers' behavior is less a result of reflective inputs and more a result of impulse, the quality of their lives suffers” (Vohs, 2006, p. 220).

VSH have recently responded to comments stating that their conceptualization of self-control can accommodate reflective self-control conflicts (see Scopelliti et al., 2020). In my humble opinion, even if we accept this notion, their conceptualization of self-control is still mute regarding the impulsive aspect of the self-control problem. In fact, while one the main goals of consumer psychology is to provide insights into when and why consumer behavior is directed by impulsive vs. reflective determinants (Hofmann et al., 2008b), VSH focus solely on deliberate and largely controlled forms of behavior. This is based on the premise that people, in their everyday lives, often act impulsively in a way that is not necessarily consistent with their declared evaluations and goals (Friese et al., 2008).

## Recommendations for Future Research Directions

Traditional research has defined self-control as the capacity of the individual to override or inhibit their competing urges, impulses, desires, and automatic or habitual responses (e.g., Muraven and Baumeister, 2000; Hagger et al., 2010). Thus, this body of literature has mostly attempted to uncover the capacity for effortful impulse inhibition, and hence has neglected automatic self-control modes (Hofmann et al., 2009; Fujita, 2011). In a somewhat different approach, VSH characterized self-control failure as a violation of superordinate long-term goals accompanied by anticipated regret. However, in my opinion, this conceptualization is also in line with the intentional notion of self-control, and hence misses the non-conscious (automated) form of self-control. Consequently, this commentary strongly recommends researchers to devote a proportionate amount of attention to non-conscious initiation of self-control including automatic goal pursuit (Bargh et al., 2001; see also Carnevale and Fujita, 2016), habitual regulatory processes, priming effects (Rebar et al., 2016), and even the effect of sleeping patterns on self-control (Williams and Poehlman, 2017). Interestingly, some researchers have even suggested that it would be more instructive to view unconscious forces as essential determinants of self-control, through which interventions can be devised that disturb the domination of impulses via habit creation or disturb the implicit associations between vice and positive affect (for a review, see Williams and Poehlman, 2017). For instance, there is evidence showing that environmental cues which are associated with motor inhibition (e.g., fearful facial expressions) can be applied to control unintentionally evoked impulses toward rewarding food objects (Veling et al., 2011). Nevertheless, this paper does not aim to underestimate controlled inputs in consumer behavior, given that both conscious and unconscious processes should be respected in consumer research (see Baumeister et al., 2017).

In addition, this paper shares VSH's general perspective that self-control researchers should ensure that participants truly experience self-control conflicts. To this end, VSH consider some crucial methodological implications to clarify how researchers can verify that participants experience self-control conflicts and how to assess them (e.g., doing pre-tests). They also highlight the role of anticipated regret as an essential indicator to correctly capture self-control conflicts and failures. However, this commentary argues that these methods do not explain how impulses emerge or how researchers can measure them. To address this issue, I encourage researchers to follow a growing body of psychological literature that aims to conceptualize self-control as a psychological process and not as a unitary phenomenon (Hofmann and Kotabe, 2012; Hofmann et al., 2012, 2014; Moayery et al., 2019a). More especially, this body of research aims to shed light on the underlying psychological process rather than focusing on the ultimate outcome variable (e.g., food consumption) (Hofmann et al., 2012, 2014). As a result, this stream of research provides a clear distinction between the strength of a given desire (i.e., impulses) and the capacity to control a desire (Hofmann et al., 2014). Interestingly, impulse formation is the starting point of this conceptualization of self-control. For instance, Moayery et al. (2019a) explained how impulses are driven by internal context (i.e., personality, homeostatic dysregulations, and habit) and external stimuli. Thus, this conceptualization provides opportunities for measuring impulses through self-report measures (e.g., Everyday Temptation Study) or implicit measures (Hofmann et al., 2012, 2014).

## Author Contributions

The author confirms being the sole contributor of this work and has approved it for publication.

## Conflict of Interest

The author declares that the research was conducted in the absence of any commercial or financial relationships that could be construed as a potential conflict of interest.
